# Denture Adhesive Utilization and Associated Factors among Dental Practitioners in the Eastern Province, Saudi Arabia

**DOI:** 10.3390/medicina59050974

**Published:** 2023-05-18

**Authors:** Mohammed M. Gad, Mayyasah O. Almusallam, Fadak H. Almarar, Haya O. Al khaldi, Alhanoof K. Aldossary, Wejdan M. Almutairi, Nawaf A. Alghamdi, Yasser S. Alssaialiy, Nawaf I. Alghamdi, Muhammad A. Nazir

**Affiliations:** 1 Department of Substitutive Dental Sciences, College of Dentistry, Imam Abdulrahman Bin Faisal University, P.O. Box 1982, Dammam 31441, Saudi Arabia; 2 College of Dentistry, Imam Abdulrahman Bin Faisal University, P.O. Box 1982, Dammam 31441, Saudi Arabia; 2170004775@iau.edu.sa (M.O.A.); 2170006198@iau.edu.sa (F.H.A.); 2170007029@iau.edu.sa (H.O.A.k.); 2170005696@iau.edu.sa (A.K.A.); 2170003668@iau.edu.sa (W.M.A.); 2160005325@iau.edu.sa (N.A.A.); 2170003897@iau.edu.sa (Y.S.A.); 2170006362@iau.edu.sa (N.I.A.); 3 Department of Preventive Dental Science, College of Dentistry, Imam Abdulrahman Bin Faisal University, P.O. Box 1982, Dammam 31441, Saudi Arabia; manazir@iau.edu.sa

**Keywords:** denture adhesives, complete dentures, dental practitioners

## Abstract

Background: Denture adhesives (DAs) enhance denture retention and stability, thus improving functions of removable prostheses. However, the adverse effects of DAs on denture foundation area were also reported. The clinical use of DAs among dentists has not been investigated in Saudi Arabia. Therefore, this study aimed to evaluate utilization of DAs and associated factors among dental practitioners in Saudi Arabia. Methods: This cross-sectional study included dental professionals practicing in both public and private sectors in the Eastern Province of Saudi Arabia. A self-administered pilot tested questionnaire was distributed among participants. The questionnaire has questions related to demographic information, knowledge and awareness, and the utilization of DAs. Bivariate and multiple logistic regression analyses were performed. Results: The study included 279 participants with a response rate of 79.03%. The majority of participants (61.6%) were below 35 years of age, males (56.6%), general dentists (57.3%), and worked in the private sector (59.9%). Less than half of the participants (39.4%) used DAs in their dental practice, and 64.5% recommended using DAs when needed. The most reported complications of DAs included inflammation (58.40%), ulcers (35.10%), and whitish color (31.20%) of denture foundation area. A vast majority (83.90%) reported that DAs improve retention of the dentures. About 55.2% of the participants were taught about DAs in their undergraduate programs, 12.5% attended continuing education, and 21.5% updated their knowledge about DAs. Multiple logistic regression showed that those who attended continuing education activities (adjusted OR = 2.41, *p* = 0.036) and updated their knowledge about DAs (adjusted OR = 4.43, *p* = < 0.001) were significantly more likely to use DAs in their dental practice. Conclusion: A minority of dental practitioners used DAs in their practices. Attending continuing education programs and updating knowledge of DAs were significantly associated with DAs utilization.

## 1. Introduction

Complete denture (CD) is considered the treatment of choice when there are no teeth remaining in one or both jaws [1] The importance of using CD has been increasing locally and globally as the number of older adults are increased, so the number of denture users is increasing, as well [2]. For older patient, CD remains the first choice due to economic factors, as well as medical conditions [3].

Retention and stability are the patient’s main concerns when it comes to CDs, and these are affected by anatomic changes in soft and hard tissues, extension of the denture’s boarders, salivary flow, and neuromuscular control [4]. When retention and stability of CDs are changed or lost, then the quality of life of CD wearers is impaired. These changes can be overcome by implant placement and ridge augmentation, but when these procedures are not possible options because of the patient’s age, medical condition, or financial status, then denture adhesives (DAs) usage can be justified as a temporary solution to have better retention, stability, and improved quality of life [5]. To ensure retention of the CDs, a broad denture foundation area is crucial [4]. 

Retention is significantly reduced if the oral mucosa has lost its original flexibility and thickness, or if the remaining alveolar ridge has been severely absorbed and has become narrow and flat [4]. Furthermore, patients’ expectations of prosthetic stability and retention are sometimes not satisfied even by the most accomplished practitioners [6,7]. Thus, denture wearers have turned towards the use of denture adhesives [8].

DAs are dental materials that are used to adhere CD to the oral mucosa [9,10,11]. DAs are available in the form of pastes, powder, or adhesive pads to be placed in the denture bearing area and eliminate the space between the denture and mucosa aiding in the denture peripheral seal [12,13]. According to the American College of Prosthodontists, DAs enhance the retention, stability, and sealing of the denture base, thus improving functions of dentures and mastication [9,11]. The DAs are reported to contribute to alleviate patients’ concerns by promoting a better fit of the new denture, leading to higher patient satisfaction [10]. The advantages of the denture adhesives are to improve the retention, to improve chewing ability, to increase stability, to provide comfort, and to reduce the accumulation of food debris below the dentures [14]. However, continuous usage of DAs can cause residual ridge resorption, as well as the development of oral diseases, such as denture stomatitis, candidiasis, and imbalance of oral flora [15]. 

Dentists should be aware of the complications of excessive use of DAs [11]. They should educate the removable denture wearers about the advantages and disadvantages of DAs and their appropriate use according to the manufacturer’s instructions to avoid any potential harm and misuse [6,10,11]. Therefore, patients will be satisfied with their DA experiences [16]. Denture wearers have been accepting DAs worldwide, regardless of the inadequate research about DAs’ long-term effects [6,11]. Most prosthodontists use DAs as a compensating material for any defects after the fabrication of dentures [14]. 

Many studies have been performed in several countries to determine the usage of DAs among dentists [5,12,13]. A study in Athens, Greece showed that 49% of prosthodontists and 61.5% of general dentists recommended the use of Das [16]. Among a group of 172 dentists from a private sector in India, 83% of general dentists, 100% of prosthodontists, and 81% of other dental specialists used the DAs as a complementary tool in their dental practice. Despite regular usage of DAs, dentists did not appear to have a thorough knowledge of these over-the-counter DAs [5]. Similarly, 44% of dentists had a lack of knowledge about the usage of DAs in Pakistan [12].

It is important to understand the knowledge and awareness about DAs among dentists in Saudi Arabia. However, there were no previous studies about dentists’ knowledge and use of DAs in the country. Therefore, the aim of the study was to evaluate utilization of DAs and associated factors among dentists in the Eastern Province, Saudi Arabia. 

## 2. Materials and Methods

### 2.1. Study Design, Participants, and Settings

A descriptive, cross-sectional survey was conducted on dental practitioners in the Eastern Province, Saudi Arabia. The cities included were Dammam, Al Khobar, Al Qatif, and Al Ahsa. The study sample calculations involved assuming 95% confidence level, ±5% precision, 0.5 degree of variability in the population, and size of the study population (*n* ≈ 3000). The Ministry of Health provides estimates of dentists working in the private and public sectors in the eastern region of Saudi Arabia [17]. An estimated sample of 353 participants was selected by using a convenience sampling technique. The practicing dentists (general dentists, specialists, and consultants) of both genders from the public and private sectors were invited to participate in the study. Dentists who work outside the Eastern Province, Saudi Arabia were excluded. 

### 2.2. Ethical Consideration

The questionnaire was distributed after obtaining institutional review board approval from Imam Abdulrahman Bin Faisal University, Dammam, Saudi Arabia (IRB-2022-02-231).Written informed consent was obtained from participants who agreed to voluntary participate in the study. The consent contained information regarding the type of the study, objectives, procedures, benefits, and duration of answering the questionnaire. Participants were assured of the confidentiality and privacy of their responses. 

### 2.3. Measurement of Study Variables

Dentists were given a self-administered questionnaire, which was based on previous studies [18,19]. The questionnaire was critically reviewed by prosthodontists and dental public health faculty at the College of Dentistry, Imam Abdulrahman Bin Faisal University. Pilot testing of the questionnaire was conducted on approximately 20 dentists; however, no changes were required. The researchers visited dental clinics and hospitals, both private and public sectors, in different cities of the Eastern province and administered hard copies of self-administered questionnaires among dental practitioners. 

The questionnaire starts with demographic information. This is followed by questions divided into three parts. The first part is composed of questions related to using DAs in dental practice (scale: yes, no), recommending DAs with normal cases (scale: Yes, as a routine practice for all patients—Yes, when needed—No, not at all), recalling patient using DAs (scale: Yes, No), teaching of DAs in undergraduate program (scale: Yes, No), attending continuing education program about DAs (scale: Yes, No), and updating knowledge about DAs (scale: Yes, No). The second part included questions to assess the knowledge and awareness of dental practitioner about DAs. Scale (Yes, very much—Yes, but only limited knowledge- and No) was used to evaluate questions related to the familiarity with DAs, chemical composition, disadvantages, guidelines by the Oral Health Foundation, and DA alternatives. For other knowledge questions, a Yes, No-, Not sure scale was used. The last part of questionnaire included questions about dentists’ knowledge regarding brands, common benefits, and complications of using DAs. 

### 2.4. Statistical Analysis 

Data analysis was performed by using the Statistical Package for Social Sciences (SPSS) software, version 22. Descriptive statistics included frequencies, percentages, means, and standard deviations. Bivariate and multiple logistic regression analyses were performed to investigate the association of independent variables (age, gender, professional qualification, place of work, years in dental practice, DAs taught in undergraduate program, attendance of a continuing education program about denture adhesives, and updating knowledge about DAs) with dependent variable (utilization of DAs). The level of statistical significance was set at 5% with a confidence interval of 95%. 

## 3. Results

A total of 353 questionnaires were administered among dental practitioners. However, 279 completed responses were returned, and the response rate of study was 79.03%. Table 1 represents the demographic data of participants. The majority of participants (61.6%) were below 35 years of age, males (56.6%), general dentists (57.3%), and worked in the private sector (59.9%). Almost sixty percent of the dentists practiced dentistry for equal or less than ten years (Table 1). 

There were less than half of the participants (39.4%) who used DAs in their dental practice. Only 2.9% of the participants recommended the use of DAs as a routine practice for all patients, 64.5% recommended using DAs when needed, and 32.6% did not recommend them at all. About one third of the participants (36.2%) recalled their patients using DAs periodically. More than half of the participants (55.2%) were taught about DAs in their undergraduate programs, 12.5% attended continuing education about DAs, and 21.5% updated their knowledge about DAs by reading books and journals (Table 2).

Most participants (72.4%) had limited knowledge about familiarity with DAs, and 59.1% were unaware of the chemical composition of DAs. Regarding the use of DAs, 63.1% of the participants agreed on the use of DAs as temporary solution. In the study, 62.7% of participants agreed that DAs can produce allergic reactions. More than half of the participants (55.6%) thought that the improper use of DAs can cause denture stomatitis, and DAs are not more effective than clinical intervention, such as relining (52.3%) (Table 3).

**Figure 1:**
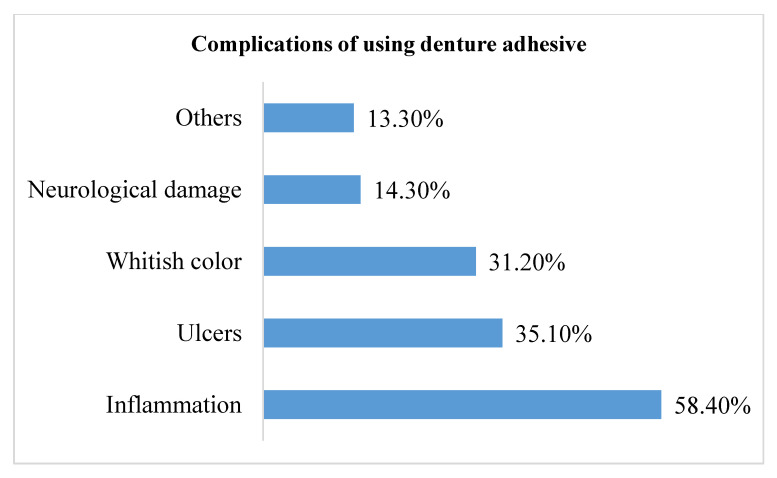
Distribution of participants’ responses about complications of using denture adhesive (*n* = 279).

The most common complication of using DAs included inflammation (58.40%), and it was followed by ulcers (35.10%) and whitish color (31.20%). Neurological damage was the least commonly reported complication (14.30%) (Figure 1). 

**Figure 2:**
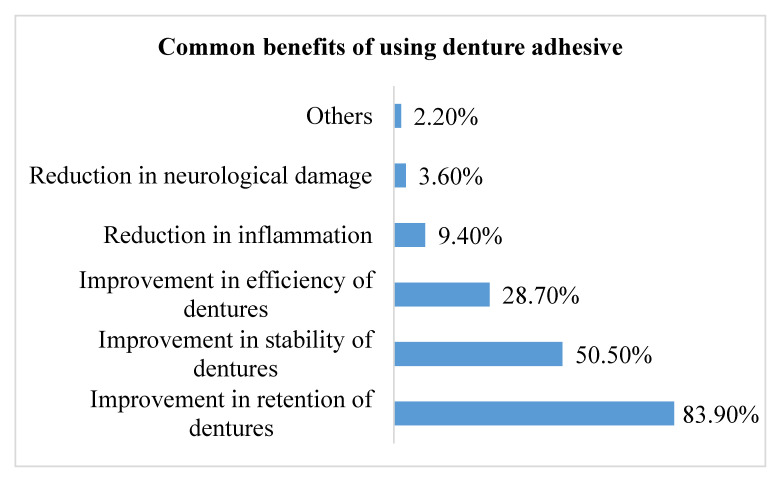
Distribution of participants’ responses about common benefits of using denture adhesive (*n* = 279).

Figure 2 represents the participants’ responses about the common benefits of using DAs. The majority of the participants (83.90%) thought that DAs will improve the retention of the dentures. This was followed by 50.50% of participants who thought that DAs improves the stability of the dentures. On the other hand, 3.60% thought that DAs reduce neurological damage.

**Figure 3:**
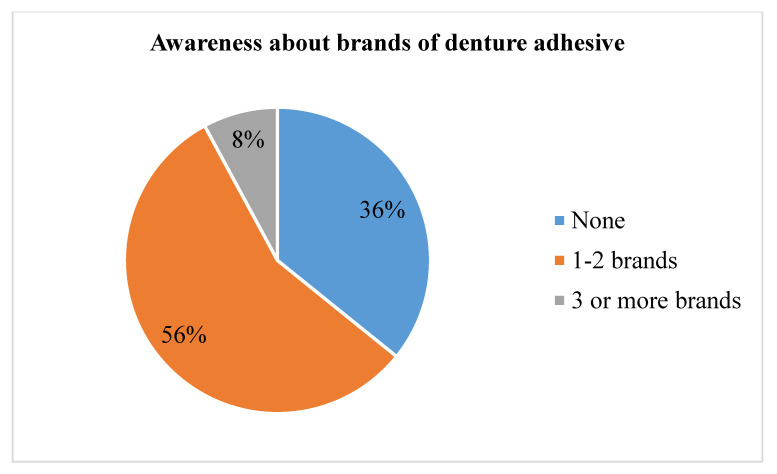
Distribution of participants’ responses about brands of denture adhesive (*n* = 279).

More than half of participants knew one to two brands of DAs, and 36% were unaware of any brand at all (Figure 3). 

Table 4 demonstrates the use of DAs in relation to the study variables in bivariate analysis. The age, gender, years in practice, attendance of continuing education program, and updating of knowledge were significantly associated with use of DAs (*p* < 0.05). 

Table 5 shows the results of multiple logistic regression analysis. The participants who attended continuing education program were 2.41 times more likely to use DAs than those who did not attend continuing education program (*p* = 0.036). Similarly, updating knowledge about DAs was significantly associated with increased odds of DA utilization (Adjusted OR = 4.43, *p*< 0.001). 

Age, gender, professional qualification, years in dental practice, place of work, teaching of DAs, attending a continuing education program, and updating DA knowledge were adjusted in the multiple logistic regression analysis.

## 4. Discussion

The purpose of this study was to measure the dentists’ utilization of DAs in Eastern Province, Saudi Arabia. The study found that 39.4% of the participants used DAs (Table 2). 

Many studies in several countries determined the usage of DAs among dentists in their dental practice [5,18,19]. A study from India showed that 83% of general dentists and 100% of prosthodontists used DAs as a beneficial adjunct in their dental practice [19]. Although DAs contains zinc, which can cause neurological disease if used in the long run, 71% of general dentists and 74% of other specialists did not have sufficient knowledge of this factor. The study reported that most general dentists and prosthodontists used DAs for CD wearers [19]. A study was performed among Nepalese prosthodontists, which reported that 48.3% prescribed DAs for their patients [20]. They believed that DAs have potential to influence the denture fit (81.7%) and provide psychological comfort (91.7%). They agreed that usage of DAs has negative impacts, such as reducing the patient’s awareness of tissue changes (65%) and reducing the recall appointment to evaluate the denture stability and retention (61.7%). 

A study of dental interns at King Saud University, Riyadh, Saudi Arabia found that 85.5% of respondents studied DAs as part of their undergraduate education [21]. In our study, 55.2% studied DAs in their undergraduate education (Table 2). The participants in our study graduated from different dental schools in Saudi Arabia and abroad, which may account for these variations. The DAs use may be increased if students are educated about the benefits of DAs in their undergraduate programs. Students must be aware that DAs are not only recommended for ill-fitting dentures, but they can be used with new dentures to improve denture functions and patient confidence [4,21]. Muneer et al. stated that DAs should be an integral part of the dental curriculum for undergraduate students [12].

In the present study, only 12% of dentists attended continuing education programs (Table 2). This low participation in continuing education programs may be attributed to the non-availability of such programs on DAs in Saudi Arabia. However, those dentists who attended continuing education in our study were significantly more likely to use DAs than those who did not (Table 5). Similarly, only 21.5% of our dentists updated their knowledge about DAs by reading books and journals, and this updating of knowledge was significantly associated with a 4.43-time greater use of DAs (Table 2 and Table 5). Dentists must be familiar with DAs to be able to recognize those patients who need them, as well as to educate them about advantages, disadvantages, and the optimum utilization of these products [21]. Mantri et al. recommended that the practitioners should attend continuing education courses about DAs to improve the patient care standard [5]. Based on the finding of the present study and the recommendation of a previous study [22], continuing education programs for DAs for dental practitioners are recommended. In agreement with Mantri et al., it was recommended that practitioners should update their knowledge by pursuing and attending continuing education courses about DAs to improve the patient care [5]. 

The majority of participants in our study (56%) were aware of one to two brands of DAs, while 36% were unaware any brand at all (Figure 3). A study conducted by Anwar et al., reported similar findings in their study. Based on the study results, the participants had limited knowledge about DA brands [23].

DAs are useful choices in cases that lack a favorable degree of stability and retention. For instance, stability and retention of CD is compromised in xerostomia, advanced ridge resorption, muscular incoordination, and deformities due to trauma or disease. Consequently, using DAs can increase retention and stability, reduce denture displacement, improve food mastication and speech, increase comfort, and achieve higher patient satisfaction [24]. In this study, the questions were used to evaluate the dentists’ knowledge about DAs, and most participants thought that DAs increase the retention of dentures, as well as improve their stability and efficacy. On the other hand, reduction in inflammation and neurological damage were the least commonly reported benefits of DAs in our study (Figure 2). This indicates a knowledge gap related to the benefits of using DAs, which could presumably reduce their application in dental practice. Thus, steps must be taken to raise awareness regarding the benefits of DAs.

Even though DAs have many advantages, they do have some disadvantages. DAs can harbor bacteria and cause oral mucosa irritation necessitate cleaning dentures thoroughly [25]. In the present study, most of the participants reported inflammation to be the most common complication, followed by ulcer and whitish discoloration on the oral mucosa (Figure 1). Evidence suggests that long-term use of DAs can cause polyneuropathies linked with spinal cord damage as a consequence of the high absorption of zinc in DAs [26,27]. Moreover, a previous study has shown that only 20% of the participants knew the adverse effects of zinc-containing DAs [28]. Similarly, another study reported that most general practitioners and other specialists were unaware of extended use of zinc-containing DAs, resulting in neurological damage [11]. In the present study, half of the participants thought that the improper use of DAs can cause denture stomatitis, and they thought that prolonged use of denture adhesives with ill-fitting dentures can cause residual ridge resorption. Taken together, it is important that appropriate measures are taken to provide comprehensive knowledge to dental practitioners regarding the possible complications of DAs and how to avoid them. 

The importance of the knowledge of using DAs includes knowing their possible complications and how to avoid them. It is essential to have good knowledge about DAs to educate patients about their dentures and products. In this study, most participants had limited knowledge about DAs (Table 3). By contrast, another study concluded that most practitioners had fair knowledge about DAs [11]. In the present study, more than half of the participants were unaware of the chemical composition of DAs. Only a few of them were aware of any disadvantages of DAs and DA guidelines by the Oral Health Foundation (Table 3). The majority of the participants agreed on the use of DAs as a temporary solution and that DAs can produce allergic reactions. Additionally, many of them did not agree that excessive use of zinc-containing DAs can cause neurological disease (Table 3). In a different study, only a fifth of the participants knew the adverse effects of zinc-containing DAs [11]. 

Our study was the first to show that the use of DAs in dental practice is affected by many factors, including low updated knowledge by reading books and journals, not attending continuing education programs, and not learning enough about DAs in undergraduate programs (Table 5). However, there are certain limitations to our study. The generalizability of the present study is limited because data were collected from the participants in the Eastern Province of Saudi Arabia. Moreover, the cross-sectional study design cannot determine cause and effect relationship among study variables. Future studies should cover wide geographical locations with an increased number of dental practitioners. In addition, a comparison between the perspectives of dental practitioners and patients regarding DAs knowledge and utilization should be considered. 

## 5. Conclusions

The findings of the present study revealed that a minority of dental practitioners used DAs in their dental practice. The dentists knew about the benefits and complications of DAs. They were aware of the impact of improper use of DAs. Few attended continuing education programs and updated their knowledge about DAs. The attendance of continuing education activities and updating knowledge about DAs were significantly associated with DA utilization.

## Figures and Tables

**Table 1:** Demographic data of study participants (*n* = 279).

Variables	Frequency (*n*)	Percent (%)
Age		
≤35 years	172	61.6
>35 years	107	38.4
Gender		
Male	158	56.6
Female	121	43.4
Professional qualification		
General dentist	160	57.3
Advanced general dentist/specialist/consultant	119	42.7
Place of work		
Government sector	112	40.1
Private sector	167	59.9
Years in dental practice/teaching:		
≤10 years	167	59.9
>10 years	112	40.1

**Table 2:** Denture adhesive use among study participants (*n* = 279).

Variables	Frequency (*n*)	Percent (%)
Used denture adhesives in your dental practice		
Yes	110	39.4
No	169	60.6
Recommended using denture adhesive with normal cases		
Yes, as a routine practice for all patients	8	2.9
Yes, when needed	180	64.5
No, not at all	91	32.6
Recalled your patients using denture adhesives periodically		
Yes	101	36.2
No	178	63.8
Taught about denture adhesives in your undergraduate program		
Yes	154	55.2
No	125	44.8
Attended a continuing education program about denture adhesives.		
Yes	35	12.5
No	244	87.5
Updated knowledge about denture adhesive by reading books/journals etc.		
Yes	60	21.5
No	219	78.5

**Table 3:** Participants’ knowledge about denture adhesives (*n* = 279).

**Items**	**Yes, Very Much** ***n* (%)**	**Yes, but Only Limited Knowledge** ***n* (%)**	**No** ***n* (%)**
Familiar with denture adhesives	53 (19.0)	202 (72.4)	24 (8.6)
Knew the chemical composition of denture adhesives	31 (11.1)	83 (29.7)	165 (59.1)
Aware of any disadvantages of denture adhesives	47 (16.8)	109 (39.1)	123 (44.1)
Aware of denture adhesives guidelines by Oral Health Foundation	25 (9.0)	71 (25.4)	183 (65.6)
Knew any alternatives to denture adhesives	37 (13.3)	60 (21.5)	182 (65.2)
**Items**	**Yes** ***n*** **(%)**	**No** ***n*** **(%)**	**Not sure** ***n*** **(%)**
Denture adhesives is considered a temporary solution	176 (63.1)	29 (10.4)	74 (26.5)
Denture adhesives can produce allergic reaction	175 (62.7)	21 (7.5)	83 (29.7)
Use of denture adhesives improves the denture foundation area	75 (26.9)	92 (33.0)	112 (40.1)
Use of denture adhesive is more effective than a clinical intervention such as relining	24 (8.6)	146 (52.3)	109 (39.1)
Denture adhesive is soluble in saliva	68 (24.4)	90 (32.3)	121 (43.4)
Improper use of denture adhesives can cause denture stomatitis	155 (55.6)	28 (10.0)	96 (34.4)
Prolonged use of denture adhesives with ill-fitting dentures can cause residual ridge resorption	137 (49.1)	44 (15.8)	98 (35.1)
Excessive use of zinc containing denture adhesives can cause neurological disease	44 (15.8)	48 (17.2)	187 (67.0)

**Table 4:** Bivariate analysis: relationship between study variables with the use of denture adhesives among study participants.

Variables	Unadjusted OR	*p*-Value
Age ≤35 years >35 years	0.50 (0.31, 0.83)	0.006
Gender Male Female	1.71 (1.05, 2.81)	0.031
Professional qualification General dentist Advanced general dentist/ specialist/ consultant	0.73 (0.45, 1.19)	0.208
Place of work Government sector Private sector	1.01 (0.62, 1.65)	0.970
Years in dental practice ≤10 years >10 years	0.61 (0.38, 1.00)	0.050
Taught about denture adhesives in your undergraduate program	1.02 (0.63, 1.65)	0.944
Attended a continuing education program about denture adhesives	3.46 (1.64, 7.29)	0.001
Updated knowledge about denture adhesives by reading books/journals, etc.	5.18 (2.78, 9.65)	<0.001

**Table 5:** Multiple logistic regression analysis: Relationship between study variables with the use of denture adhesives among study participants.

Variables	Adjusted OR	*p*-Value
Age ≤35 years >35 years (Reference category)	0.46 (0.19, 1.09)	0.078
Gender Male Female (Reference category)	1.30 (0.74, 2.29)	0.364
Professional qualification General dentist Advanced general dentist/specialist/consultant (Reference category)	1.21 (0.65, 2.28)	0.548
Place of work Government sector Private sector (Reference category)	0.97 (0.55, 1.71)	0.920
Years in dental practice ≤10 years >10 years (Reference category)	1.22 (0.51, 2.93)	0.658
Taught about denture adhesives in your undergraduate program Yes No (Reference category)	1.11 (0.65, 1.91)	0.705
Attended a continuing education program about denture adhesives Yes No (Reference category)	2.41 (1.06, 5.46)	0.036
Updated knowledge about denture adhesives by reading books/journals etc. Yes No (Reference category)	4.43 (2.31, 8.48)	<0.001

## Data Availability

The data are available upon request via email or phone to the corresponding author.
